# Life-Threatening Rupture of a False Aneurysm after Femoral Arterial Catheterization: Unexpected Delay after a Common Procedure

**DOI:** 10.1155/2013/403507

**Published:** 2013-05-22

**Authors:** Julie Renner, Pierre Pasquier, Elisabeth Falzone, Faye Rozwadowski, Stéphane Mérat

**Affiliations:** ^1^Department of Anesthesiology and Intensive Care, Bégin Military Teaching Hospital, 69 avenue de Paris, 94160 Saint-Mandé, France; ^2^Intensive Care Unit, Bégin Military Teaching Hospital, 94160 Saint-Mandé, France; ^3^Intensive Care Unit, Percy Military Teaching Hospital, 92140 Clamart, France; ^4^Naval Branch Health Clinic, United States Navy, Lakehurst, NJ 08733-5006, USA

## Abstract

We report the case of a 79-year-old patient who presented with a femoral iatrogenic pseudoaneurysm of delayed and unusual onset with immediately life-threatening massive bleeding. Ultrasound is the method of choice for the diagnosis of pseudo aneurysm. If there is not spontaneous closure, ultrasound-guided compression repair, minimally invasive percutaneous treatments, and surgical repair are the three therapeutic options.

## 1. Summary

A 79-year-old female patient was admitted to the intensive care unit for acute respiratory failure on day 1. 

Pertinent medical history included chronic obstructive pulmonary disease requiring long-term oxygen and corticosteroid therapies, atrial fibrillation, and pacemaker insertion. Her multiple medications included anticoagulation with fluindione, amiodarone, furosemide, rabeprazole, prednisolone, terbutaline, budesonide, and salbutamol.

The patient deteriorated, was intubated, and mechanically ventilated for 2 days. She developed pneumonia with secondary septic shock, managed with norepinephrine for 48 hours and antibiotic therapies (piperacillin tazobactam and amikacin), as well as heart failure with required inotropic support for 72 hours. Continuous cardiac output using pulse contour analysis was measured by the PiCCO plus (PULSION Medical Systems, Munich, Germany). An arterial pressure line was inserted, using the Seldinger technique, into the right femoral artery (5 F, 20 cm long thermistor-tipped arterial catheter PV2015 L20-A; Pulsion Medical Systems, Munich, Germany) and connected to the cardiac output monitor for 5 days. The catheter removal was followed by manual compression for 15 minutes and bed rest.

The patient was discharged in stable condition on day 5, from the ICU to the cardiology unit. 

On day 17, the patient experienced severe sharp pain in her right groin. Physical examination revealed hypotension (70/50 mmHg) and a voluminous mass in the right groin. The patient was transferred to the intensive care unit.

On admission to intensive care unit, the mass in the right groin was indurate, pulsatile, and growing, without sign of neither nerve compression nor ischemia.

Blood sample analysis was the following: haemoglobin 5.4 g/L, platelets 103 × 10^9^/L, prothrombin time (PT) 21.6 seconds, INR 2, activated partial thromboplastin time (aPTT) 1.57 second, fibrinogen 4.2 g/L, pH 7.26, partial pressure of oxygen 164 mmHg, partial pressure of carbon dioxide 39 mmHg, HCO_3_ 17.7 mmol/L, and lactates 1.9 mmol/L. 

Hemodynamic instability and a fall in haemoglobin indicated massive haemorrhagic shock of the right femoral artery lesion about 12 days after the removal of the catheter.

Medical management included compressive dressing, anticoagulation reversal (vitamin K and prothrombin complex concentrates), hypotensive resuscitation, and transfusion of 6 units of red blood cells (RBC) and 4 units of fresh frozen plasma (FFP). 

**Figure 1:**
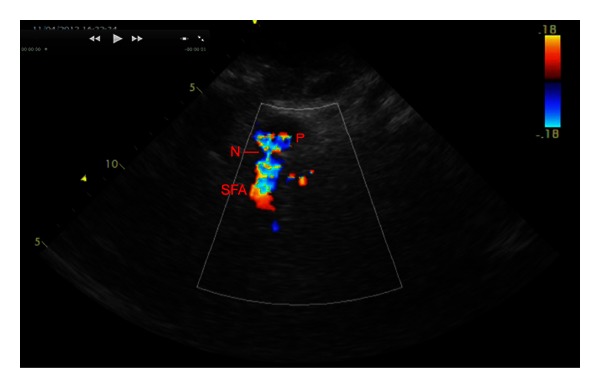
Ultrasound image: pseudoaneurysm sac (P) communicates via a neck (N) with the superficial femoral artery (SFA). Colour Doppler demonstrates flow within the pseudoaneurysm.

**Figure 2:**
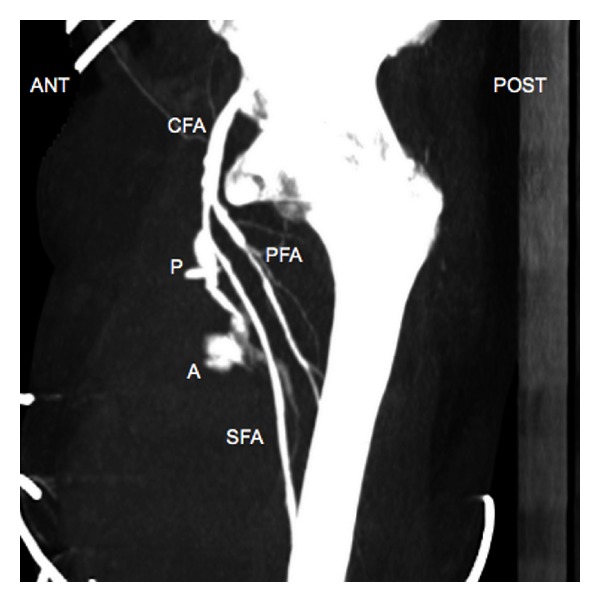
Sagittal computed tomography angiography image of the right groin demonstrates a large pseudoaneurysm (P) communicating with the right superficial femoral artery (SFA) through a pseudoaneurysm neck (N). Common femoral artery (CFA). Profunda femoris artery (PFA). Active bleeding (A).

**Figure 3:**
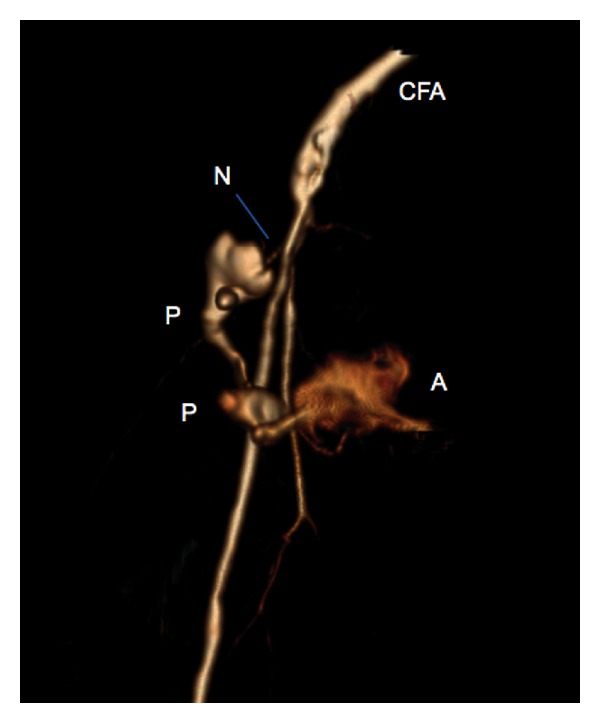
3D view computed tomography angiography image of the right groin: large pseudoaneurysm (P) communicating with the right superficial femoral artery through a pseudoaneurysm neck (N). Femoral common artery (CFA). Active bleeding (A).

Doppler ultrasound confirmed internal blood flow (Figure 1), but the anatomy was not well defined. Contrast-enhanced computed tomography was performed, confirming a large haematoma (23 × 20 × 10 cm, so 2.3 L) with an arterial breach of the right superficial femoral artery and no arteriovenous fistula nor retroperitoneal localization (Figures 2 and 3). 

The patient underwent emergency haemostatic surgery due to hemodynamic instability. During surgical examination, a right femoral artery pseudoaneurysm rupture was noted at the puncture site of the original arterial catheterization. The arterial axis was normal with no aneurysmal dilatation. Surgical haemostasis (wound closure, exclusion of the false aneurysm, and use of TachoSil (Absorbable Fibrin Sealant Patch)) and management of coagulopathy, including transfusion of RBCs, FFPs, fibrinogen, and prohaemostatic therapy, permitted rapid clinical improvement.

Norepinephrine was stopped on day 18 and the antithrombotic therapy resumed on day 20 without any more complications. Perioperative bacterial cultures remained sterile. 

The patient came back home on day 57.

## 2. Discussion

Indwelling arterial catheters are used in critically ill patients for continuous hemodynamic monitoring and multiple blood samplings, especially arterial blood gases [1, 2].

Arterial cannulation is a commonly performed procedure considered relatively safe; few major complications occurring in less than 1% of the cases [1]: haematoma at the puncture, sepsis, pseudoaneurysm formation, ischemic damage, arteriovenous fistula, arterial dissection, and catheter migration [3, 4].

False aneurysm, also called pseudoaneurysm, is a collection of blood formed as a result of a vascular wound and retained in the tissues surrounding the vessel breached. The resulting pseudoaneurysm consists of a perfused sac, the false lumen, connected to the femoral artery by a neck [3]. 

The incidence of after procedure pseudoaneurysm is increasing with the development of cardiac or peripheral vascular procedures [3, 4]. Its incidence varies from 0.48% [5] for diagnostic procedures to 10% for therapeutic procedures, depending on length, complexity of procedure, and the size of involved cannulas. Moreover, *S. aureus* infection and persistent bacteraemia after catheter removal are a major risk factor for pseudoaneurysm formation [5]. Many other factors are involved [3, 4]: catheterization of both artery and vein or catheterization of superficial/deep femoral artery for procedure-related risk factors, female gender, age over 60 years, obesity, calcified arteries, anticoagulant/thrombolytic/antiplatelet agents, and haemodialysis for patient-related risk factors. Low femoral puncture and inadequate compression post-procedure are also associated with postprocedure pseudoaneurysm formation.

The average time of pseudoaneurysm onset varies from 5 to 6 days after catheter removal [4, 5]. Here, we report a later onset of the pseudoaneurysm, 12 days after catheter removal, which was especially unexpected. However, the diagnosis was fast since the patient presented with marked pain and pulsatile haematoma, and new thrill in the groin, a classic presentation [3, 4]. Ultrasound (US), with sensitivity between 94 and 97%, is the gold standard to diagnose pseudoaneurysm [3]. US findings can include swirling color flow seen in a mass separate from the affected artery, colour flow within a tract leading from the artery to the mass consistent with pseudoaneurysm neck, and a typical “to and fro” Doppler waveform in the pseudoaneurysm neck [4]. In our case, US findings were equivocal and the anatomy was not well defined: contrast-enhanced computed tomography was of value.

Complications of pseudoaneurysms include distal embolization, manifestations due to mass effect, and rupture leading to catastrophic bleeding. Scheer et al. identified 11 studies published over 23 years, with a total of 3899 reviewed cases of femoral artery cannulation for hemodynamic monitoring. Pseudoaneurysm formation occurred in six patients (mean incidence 0.3%), bleeding (generally minor) was observed in five patients (mean incidence 1.58%). Only one patient developed an infected haematoma and needed blood transfusion [6] and another patient eventually died from massive retroperitoneal bleeding [7]. 

There is no standardized management of the postprocedure false aneurysms. In most cases, the natural history of these false aneurysms is spontaneous closure. Several therapeutic strategies have been developed to treat postprocedure false aneurysm [4]: ultrasound-guided compression repair, minimally invasive percutaneous treatments (thrombin injection, coil embolization, and insertion of covered stents), and surgical repair [3]. In our case, absolute indications for surgical repair were hemodynamic instability and rapid expansion [3, 4]. 

In summary, We report the case of a 79-year-old patient who presented a femoral iatrogenic pseudoaneurysm of delayed and unusual onset with immediately life-threatening massive bleeding [5]. 

Based on this significant experience, we could make the following recommendations for prevention of postcatheterization false aneurysms:

1. Nontraumatic puncture of the common femoral artery, ideally ultrasound guided [3, 4]. Ultrasound-guided vascular puncture reduces the number of failed puncture and detects anatomic variations and lesions. It has been showed to be an easy and safe technique [2].
2. a size of introducer between 5 and 7 F;
3. manual or mechanical compression of 15–20 minutes followed by bed rest and compressive dressing;
4. in case of coagulopathy or thrombolytics/antiplatelet agents/anticoagulants treatment: arterial puncture closing devices (vascular closure system as “plug” of collagen or sophisticated equipment to close the arterial puncture site) can be used. However, it is associated with a significant increase in the risk of vascular complications [8, 9].
